# Applying Sydney Triage to Admission Risk Tool (START) to improve patient flow in emergency departments: a multicentre randomised, implementation study

**DOI:** 10.1186/s12873-024-00956-5

**Published:** 2024-03-07

**Authors:** Saartje Berendsen Russell, Radhika V Seimon, Emma Dixon, Margaret Murphy, Matthew Vukasovic, Nicole Bohlken, Sharon Taylor, Zoe Cooper, Jennifer Scruton, Nitin Jain, Michael M Dinh

**Affiliations:** 1 Emergency Department, RPA Green Light Institute, Royal Prince Alfred Hospital, Missenden Road, 2050 Camperdown, NSW Australia; 2 Emergency Department, Westmead Hospital, Westmead, NSW Australia; 3 Emergency Department, Concord Repatriation General Hospital, Concord, NSW Australia

**Keywords:** Emergency, Patient flow, Triage, Decision support

## Abstract

**Background:**

To determine the effectiveness of applying the Sydney Triage to Admission Risk Tool (START) in conjunction with senior early assessment in different Emergency Departments (EDs).

**Methods:**

This multicentre implementation study, conducted in two metropolitan EDs, used a convenience sample of ED patients. Patients who were admitted, after presenting to both EDs, and were assessed using the existing senior ED clinician assessment, were included in the study. Patients in the intervention group were assessed with the assistance of START, while patients in the control group were assessed without the assistance of START. Outcomes measured were ED length of stay and proportion of patients correctly identified as an in-patient admission by START.

**Results:**

A total of 773 patients were evaluated using the START tool at triage across both sites (Intervention group *n* = 355 and control group *n* = 418 patients). The proportion of patients meeting the 4-hour length of stay thresholds was similar between the intervention and control groups (30.1% vs. 28.2%; *p* = 0.62). The intervention group was associated with a reduced ED length of stay when compared to the control group (351 min, interquartile range (IQR) 221.0–565.0 min versus 383 min, IQR 229.25–580.0 min; *p* = 0.85). When stratified into admitted and discharged patients, similar results were seen.

**Conclusion:**

In this extension of the START model of care implementation study in two metropolitan EDs, START, when used in conjunction with senior early assessment was associated with some reduced ED length of stay.

**Supplementary Information:**

The online version contains supplementary material available at 10.1186/s12873-024-00956-5.

## Introduction

Emergency Departments (ED) overcrowding occurs due to excessive numbers of patients and results in delays in assessing or treating patients in ED [1]. Access block, a major contributor to ED overcrowding occurs when an ED patient, needs to be admitted to an in-patient specialist service hospital ward and requires an ED bed because of a lack of in-patient ward bed capacity. Consequently, patients often remain for prolonged periods in the ED until an in-patient hospital ward bed becomes available. ED overcrowding can be particularly detrimental for patients who need time-critical interventions to effectively treat emergency conditions such as acute stroke, [2] acute respiratory failure, [3] and septic shock [4]. Further, numerous studies have demonstrated adverse patient outcomes resulting from ED overcrowding, including increased mortality during hospital stay, particularly for older patients susceptible to delirium and falls, increased clinician decision-making time, increased medication errors, increased in-patient length of stay, increased hospital costs, delayed treatment, poorer quality of care and complications [5–10]. The current health response to the novel coronavirus (COVID-19) pandemic has also drawn attention to the importance of improving the management of patient flow and overcrowding to avoid overwhelming health services and provide timely care to patients.

We developed the Sydney Triage to Admission Risk Tool (START) and conducted a single-centre implementation study on START, using a matched case control sample of patients. In this small study we showed that the intervention group model of care was associated with a significant reduction of ∼2 h in ED length of stay 301 min (interquartile range, IQR 225–397 min) vs. 423 min (IQR 297–587 min) *P* < 0.001) and the proportion of patients meeting the 4-hour length of stay thresholds in the ED doubled in the intervention group, from 19 to 42% (*P* < 0.001)^11^.

The aim of the present study is to test START in combination with a senior early assessment model of care and determine its effectiveness with respect to ED length of stay and accuracy of decision making in a multicentre trial at two different hospitals in New South Wales, Australia.

## Methods

### Design and setting

This was a multicentre study of a model of care implementation. The study was conducted at two tertiary Hospital Emergency Departments in metropolitan Sydney, Australia, with ∼ 40,000 and ∼ 80,000 ED presentations per year in each hospital. The smaller hospital is a mixed ED seeing adults and paediatrics and the second an adult ED.

### Patient population

This study used a convenience sample of adult patients presenting to the ED during business hours, Monday to Friday, between July and September 2021. The days were based on research investigator availability (two days per week per site), and randomised to control or intervention by allocation concealment methods. The patients arriving on those days were selected consecutively. Immediately life-threatening presentations (trauma calls, stroke calls, LifeNet (acute myocardial infarction) and cardiac arrest calls, transfers from other hospitals, expected admissions and those brought in by police were excluded. The research investigators observed the complete triage interactions between the patient and triage nurse in both the control and intervention groups and completed the START score data collection form in real time. At the end of triage, the score was calculated and conveyed to the Admitting officer and or Navigator either by phone or in person. After the START score was passed on, the research investigator observed the next whole triage, completing as many consecutive eligible triage observations over the shift as possible. If the research investigator was not able to observe the whole triage, that patient was not included. The research investigator was not involved in the triage process in any way.

### Group allocation

Patients were allocated based on day of arrival with each day randomised into the intervention or control group days. This was done using sealed envelopes as an allocation concealment method and determining a schedule for data collectors to follow. All clinicians were aware of the study that was initiated at triage, but not the specific process or outcomes and inpatient treating teams were not involved.

### Intervention

START was derived and internally validated in 2016^9^ and a pilot study completed in 2019^11^ (Supplement 1). Researchers from the original START pilot implementation study met with stakeholders at the two EDs and trained the research investigators (i.e. ED senior nurses) in the tool and study protocol. Each site then engaged with their staff and informed them of the study, as senior early assessment and team-based models of care were already in place. The study compared patients who were admitted, after presenting to both EDs, and were assessed using the existing senior ED clinician assessment with the assistance of START (intervention) and without the assistance of START (control).

Patient’s triage interactions were observed by the research investigator at each site to determine the likelihood of in-patient admission on study days using START (i.e., START score > 16 points). ED short-stay admission, an inpatient ward managed by ED that admits patients with an expected length of stay < 24 h, were not included and were removed retrospectively. The disposition to short stay would be unknown at the point of triage when the START score is calculated. Short stay patients were not included in the study as we were looking at in-patient admissions to the hospital. This occurred in parallel with usual triage practices.

For those patients that score > 16 points on START, the research investigator notified the senior ED clinician in charge of these patients. The role of the senior ED clinician was to decide, based on their brief senior early assessment and START score, whether the patient would in fact be admitted to an inpatient hospital ward and which in-patient specialist service the patient would most likely be admitted under. This decision was then communicated to hospital bed managers (by icons fired in the electronic medical records (eMR) and/or by the ED Navigator or NUM) Existing patient flow unit processes would then follow in terms of searching for or allocating an inpatient bed based on this information, whilst full assessment including investigations, consultations and initial management were simultaneously being completed in ED.

### Controls

The control group were patients who were admitted under standard practices in the ED by clinicians, without the assistance of START by the research investigator. For a typical ED patient the ED models of care, typically commences with triage, followed by an ED nursing assessment, junior doctor medical assessment and may involve review by an ED registrar and /or ED Consultant, followed by investigations, consultation with other services and review by in-patient teams, before a decision on disposition is formally made. This process usually takes hours depending on the complexity of a patient’s medical history.

The designated research investigator scored the triage encounter using START, for descriptive purposes only; however, results of the risk scoring were not be made known to the senior ED clinician or included in the electronic clinical notes.

### Study outcomes

The primary outcomes were total ED length of stay and the proportion of patients with an ED length of stay less than four hours. The secondary outcomes were time to final disposition (defined by the time that “admission ready” icons were fired in the ED tracking system– time to disposition was calculated by subtracting disposition time by triage time) and the proportion of intervention patients who were correctly assigned as an admission.

### Statistical analyses

Age, gender, triage category, ED length of stay, disposition and admitting specialty are all variables collected through existing patient information systems. Descriptive statistics with contingency tables were used to evaluate baseline characteristics of cases undergoing senior early assessment with the aid of START (intervention) and control groups. Categorical variables collected through existing patient information systems included age, gender, triage category, ambulance arrival, presenting problem were tested using Chi-square tests. Mann-Whitney U tests were used to test continuous variables, such as ED median length of stay (in minutes), median time for disposition (in minutes) between groups. Logistic regression was fitted using the START score to predict admissions and was evaluated using area under curve of receiver operator characteristic (AUC ROC). Analyses were performed using SPSS Version 26.

A power calculation was performed based on the intention to implement the study at 3 sites, unfortunately due to COVID the study was only conducted at 2 sites. We calculated that a target sample size of 1,200 patients would provide enough power to detect a 10% improvement in proportion of patients staying in ED less than four hours with a power of 0.80 and a 2-sided α level of 5%, as seen in previously published derivation study [9]. 

### Ethics

Ethical approval for the research was received from the Sydney Local Health District Research Ethics Committee (Reference Number 2019/ETH12088). The research investigators had no direct interactions with patients apart from observing them being triaged. Therefore, a waiver for informed consent was sought and approved by the Sydney Local Health District Research Ethics Committee. The trial was conducted in accordance with the Good Clinical Practice guidelines.

## Results

### Study population

Participants were recruited between July and September 2021, and 773 patients were randomised into the study. Of the 773 patients, 355 patients were randomised to the intervention group and 418 patients were randomised to the control group. Baseline characteristics, including age, gender, triage category, ambulance arrival status and presenting problem were all similar between the intervention and control groups (Table 1). START scores between groups were also comparable.

### Outcomes

The intervention group was associated with a reduced ED length of stay when compared to the control group (351 min, interquartile range (IQR) 221.0–565.0 min versus 383 min, IQR 229.25–580.0 min; *p* = 0.62). When stratified into admitted and discharged patients, similar results were seen for the admitted patients, while there was no difference in ED length of stay for the discharged patients. The proportion of patients meeting the 4-hour length of stay thresholds was similar between the intervention and control groups (30.1% vs. 28.2%; *p* = 0.62). When stratified into admitted and discharged patients, similar results were seen (Table 2).

The START score when applied to the data had an AUC ROC of 0.77 (95% CI 0.74–0.80). Using the recommended START score of 16, the START score was able to predict patients’ admission with 87% sensitivity and 45% specificity.

## Discussion

The present study was conducted to evaluate an implementation of a model of care designed to facilitate patient flow in ED in different EDs than previously tested. The model of care involved utilising START, a validated clinical analytics tool in conjunction with senior early assessment in ED. The tool is designed to alert senior ED clinicians to patients likely to need in-patient admission and to facilitate bed management earlier in the patient’s journey. The driver of this study was to establish the utility of START across other ED settings and ascertain if it could reduce ED length of stay in other ED sites.

We found that a model of care involving a START score category of ‘likely admission’ was associated with improved time to disposition. In such patients, the median length of stay was 32 min less than control patients, although not statistically significant, it is still significant in managing ED patient flow and improving bed availability for a larger number of patients. The findings are promising enough to do further implementation studies.

The authors acknowledge that the findings of this study differ when compared to the original study that showed an almost 2-hour reduction in length of stay and note the contrast could be attributed to differences in population, staffing and models of care at individual sites and whilst not statistically significant, there are still several important implications for the current findings combined with the original single site study findings.

Firstly, many analytics and risk prediction tools are in use clinically [12–14], and several have reported admission prediction models [15–18], however our studies apply a clinical analytics tool in real-time. Additionally, the use of this tool to support senior early assessment is based on the idea that expedited bed management strategies could be data driven, automated and commence earlier in the patient’s journey occurring at the same time as their ED assessment and treatment. This differs to standard practice where bed finding occurs after assessment and after an in-patient team has been nominated.

There are also other reasons that the tool may not reduce a patient’s total length of stay while still shortening the time to disposition decision time. These reasons including access block, rostering, variation in clinical practice, models of care and COVID-19 impacts. For example, there could be variation in the application of admissions policy site to site based on facility culture and established workflows as well as the reality that there will also be many times when access block occurs, and an appropriate bed will not be available regardless of the lead time provided through senior early assessment and START application.

The impact of COVID-19 (Delta wave) on each ED and the introduction of new COVID-19 models of care that provided alternative streaming from triage and/or alternative admission pathways for patients presenting with confirmed or suspected COVID-19 and the introduction of COVID-19 wards may also contribute to the current results. It is also noted that ED attendances and admissions varied during COVID-19 and were influenced by fear, lock-downs and access to primary health [19, 20]. Additionally, due to COVID-19, the study was only conducted at 2 sites and was therefore very much underpowered, although still informative.

Other limitations to this study include the imbalance of patient numbers in each group, the study being conducted at the different sites on different days relating to research investigator rostering and therefore different consultants being involved on the days in which the model was tested. However, this also reflects ‘normal business practice’ with different consultants and support staff being rostered in ad hoc patterns in EDs routinely. There were limitations on enrolment of patients in that, the days were based on research investigator availability (two days per week per site) and if the research investigator was unable to observe the whole triage, that patient was not included. Other confounders include workflow styles of individual consultants and openness to model of care changes. Differences in day of the week and time of day of presentations may have also accounted for some of the differences in ED length of stay.

While the implications of the low specificity of this study need to be considered, the researchers would like to further evaluate this model of care using either a patient level randomised control study or cluster randomised control study at a number of different sites to confirm external validity. Funding has been secured for a translational research study to implement this at scale, and further refine the tool using machine learning with linked datasets.

Future trials would like to include the incorporation of the START score into existing patient information systems because most of the variables that determine the START score can be automatically calculated or observed at the point of triage. Opportunities exist for this clinical analytics tool to be activated in real time within existing electronic patient information systems. Potential changes to the triage process would need to be considered to facilitate this and additional studies are needed to establish which groups of patients would benefit most from the extra intervention required at triage. However, future applicability for this tool would need to be investigated using a cluster randomised controlled study to evaluate the effect START may have on expediting admissions processes.

In conclusion, in this multicentre implementation study, the use of START, a clinical analytics tool to support senior early assessment in ED was associated with a significant reduction in time to ED disposition but not associated with a significant reduction in ED length of stay. These findings will form the basis of further implementation studies.

**Table 1:** Baseline characteristics of cases undergoing senior early assessment with the aid of the Sydney Triage to Admission Tool (START; intervention) and control groups

Variable	Intervention (*n* = 355) n (%)	Control (*n* = 418) n (%)	P-Value
Age (years)			0.92
16–19	4 (1.1)	8 (1.9)	
20–39	65 (18.3)	76 (18.2)	
40–59	89 (25.1)	100 (23.9)	
60–79	114 (32.1)	139 (33.3)	
> 80	83 (23.4)	95 (22.7)	
Gender (%)			0.09
Female	193 (54.4)	199 (47.6)	
Male	155 (43.7)	209 (50.0)	
Triage Category (%)			0.42
5	4 (1.1)	2 (0.5)	
4	61 (17.2)	61 (14.6)	
3	150 (42.3)	167 (40.0)	
2	129 (36.3)	175 (41.9)	
1	11 (3.1)	13 (3.1)	
Ambulance arrival (%)	144 (40.6)	182 (43.5)	0.40
Actual disposition (%)			0.96
Admitted	219 (61.7)	256 (61.2)	
Discharged	129 (36.3)	152 (36.4)	
START score (median IQR)	22 (15–29)	22 (17–29)	0.90
Presenting Problem (%)			0.49
Abdominal, Gastrointestinal	36 (10.1)	42 (10.0)	
Cardiovascular	52 (14.6)	74 (17.7)	
General symptoms	58 (16.3)	58 (13.9)	
Febrile illness	9 (2.5)	11 (2.6)	
Injury	55 (15.5)	71 (17.0)	
Respiratory	41 (11.5)	31 (7.4)	
Musculoskeletal	13 (3.7)	14 (3.3)	
Neurological	38 (10.7)	40 (9.6)	
Mental health	12 (3.4)	12 (2.9)	
Toxicological	1 (0.3)	4 (1.0)	
ENT/eye/head/neck	6 (1.7)	11 (2.6)	
Genitourinary	9 (2.5)	15 (3.6)	
Social	1 (0.3)		
Endocrine	1 (0.3)	2 (0.5)	
Obstetrics, gynaecology	3 (0.8)		
Skin, allergy	7 (2.0)	14 (3.3)	
Other medical	13 (3.6)	18 (4.3)	

IQR, interquartile range; ENT, ear nose throat

**Table 2:** Study outcomes for all patients and stratified into patients admitted and discharged

Outcome	Intervention *n* = 355	Control *n* = 418	P-Value
Total ED length of stay– all patients (mins, median IQR)	351 (221.0–565.0)	383 (229.25–580.0)	0.62
Total ED length of stay– admitted patients only (mins, median IQR)	441 (280.0–701.0)	469.5 (282.5–774.0)	0.59
Total ED length of stay– discharged patients only (mins, median IQR)	264 (184.5–400.0)	258.0 (181.0–413.5)	0.89
ED Length of stay < 4 h (%) - all patients	107 (30.1)	118 (28.2)	0.62
ED Length of stay < 4 h (%) - admitted patients only	45 (20.5)	48 (18.8)	0.26
ED Length of stay < 4 h (%) - discharged patients only	59 (45.7)	65 (42.8)	0.06
Time to disposition - admitted patients only (mins, median IQR)	136 (29.0–230.0)	182.0 (104–263)	< 0.001

ED, emergency department; IQR, interquartile range

### Electronic supplementary material

Below is the link to the electronic supplementary material.

Supplementary Material 1

## Data Availability

The datasets used and/or analysed during the current study are available from the corresponding author on reasonable request.
