# Supplementary material for: Applying Sydney Triage to Admission Risk Tool (START) to improve patient flow in emergency departments: a multicentre randomised, implementation study
Source: BMC Emerg Med. 2024 Mar 7;24:39. doi: 10.1186/s12873-024-00956-5 (PMC10921805; doi:10.1186/s12873-024-00956-5)
Supplement: Supplementary file 1 — Supplementary Material 1 [file 12873_2024_956_MOESM1_ESM.docx]

**Supplement 1:** START Tool

| **Variable** | **Risk Score** | **Variable** | **Risk Score** |
| --- | --- | --- | --- |
| **Age** |  |  |  |
| 16-19 years | 0 | **Decreased mobility or frailty** | +5 |
| 20-39 years | +1 | **Multiple or major comorbidities** | +5 |
| 40-59 years | +3 | Referred by GP/specialist with diagnosis and or needing admission | Y/N |
| 60-79 years | +6 | Significant ED overcrowding (>3 ambulances or TOC trolleys waiting to be offloaded or >25 patients waiting to be seen) | Y/N |
| ≥80 years | +9 | Other factors….please specify |  |
| **Ambulance arrival** | +4 | **TOTAL START SCORE** |  |
| **Triage Category** |  | \|  \| Risk score range \| Stream / location \| \| --- \| --- \| --- \| \| Very likely discharge \| <5 \| WR/RAFT/refer to ambulatory care or HITH \| \| Likely discharge \| 5-10 \| RAFT, EDSSU results/transport pending \| \| Indeterminate \| 11-15 \| Decision pending clinician assessment \| \| Likely admission \| 16-25 \| Look for inpatient bed, work up for admission, notify EDAO and NUM \| \| Very likely admission \| >25 \| Book inpatient bed and inform bed management and inpatient team of arrival \|   **Examples of major co-morbidities:**  In chemotherapy/cancer treatment  Renal or liver transplant or haemodialysis  Congenital heart disease  Cystic fibrosis | |
| 1 | +24 |  |  |
| 2 | +16 |  |  |
| 3 | +11 |  |  |
| 4 | +5 |  |  |
| 5 | 0 |  |  |
| **Admission within 30 days** | +3 |  |  |
| **Hour of presentation** |  |  |  |
| 08:00 – 17:59 | +1 |  |  |
| 18:00 – 22:59 | 0 |  |  |
| 23:00 – 07:59 | 0 |  |  |
| **Presenting problem** |  |  |  |
| Abdominal, gastrointestinal | +2 |  |  |
| Cardiovascular | -3 |  |  |
| General symptoms | 0 |  |  |
| Febrile illness | +3 |  |  |
| Injury | -4 |  |  |
| Respiratory | 0 |  |  |
| Musculoskeletal | -3 |  |  |
| Neurological | -1 |  |  |
| Mental health | -2 |  |  |
| Toxicological | -2 |  |  |
| ENT/eye/head and neck | -6 |  |  |
| Administrative | -3 |  |  |
| Genitourinary | -1 |  |  |
| Social | +1 |  |  |
| Endocrine | 0 |  |  |
| Obstetrics, Gynaecology | -3 |  |  |
| Skin, allergy | -2 |  |  |
| Other medical | +5 |  |  |
